# Multidimensional assessment of cognitive function in community-dwelling older adults: fNIRS dual-task assessment and nutritional evaluation in Japanese salons

**DOI:** 10.3389/fpubh.2026.1770437

**Published:** 2026-06-30

**Authors:** Nobuko Shimizu, Noboru Hasegawa, Takako Yamada, Tomohiro Umemura, Minatsu Kobayashi, Mayumi Kato, Kyosuke Yorozuya, Antonio Oliveira Nzinga Rene, Masahiro Matsunaga

**Affiliations:** 1 Faculty of Nursing, Toyama Prefectural University, Toyama, Japan; 2 Faculty of Rehabilitation, AICHI Medical College of Rehabilitation, Kiyosu, Aichi, Japan; 3 School of Health Sciences, Bukkyo University, Kyoto, Japan; 4 School of Health and Sport Sciences, Chukyo University, Toyota, Aichi, Japan; 5 Faculty of Home Economics, Otsuma Women's University, Chiyoda-ku, Tokyo, Japan; 6 Faculty of Information Engineering, Toyama Prefectural University, Toyama, Japan; 7 Faculty of Human Health, Aichi Toho University, Nagoya-shi, Aichi, Japan

**Keywords:** cognitive function, community salon (senior salon), community-based prevention, dual-task, fNIRS, mild cognitive impairment, nutrition, older adults

## Abstract

**Introduction:**

Motor–cognitive dual-task performance is closely related to age-related changes in cognitive and motor function. Community-based approaches combining cognitive screening with neurophysiological and nutritional assessment may help characterize cognitive health in older adults. This study explored the feasibility of combining prefrontal oxygenated hemoglobin (Oxy-Hb) responses measured using functional near-infrared spectroscopy (fNIRS) during a seated motor–cognitive dual-task paradigm with cognitive screening and nutritional evaluation among older adults attending community salons.

**Methods:**

A total of 127 community-dwelling older adults attending local salons were included. Cognitive function was assessed using the Montreal Cognitive Assessment–Japanese version (MoCA-J) and the Mini-Mental State Examination. Nutritional intake and body composition were evaluated using a food frequency questionnaire and bioelectrical impedance analysis. Prefrontal Oxy-Hb responses were recorded using an 8-channel fNIRS system during a seated motor–cognitive dual-task condition. Exploratory hierarchical multiple regression analyses examined associations between MoCA-J scores and Oxy-Hb responses. Age and sex were entered as covariates, and all eight fNIRS channels were entered simultaneously using the forced-entry method. Multiple comparisons across fNIRS channel predictors were addressed using Benjamini–Hochberg false discovery rate correction.

**Results:**

Approximately 60.3% of participants scored below the MoCA-J cutoff, suggesting possible cognitive decline based on screening. For the MoCA-J total score, the overall regression model was significant, and adding the eight fNIRS channels significantly improved model fit beyond age and sex. After false discovery rate correction, Ch6 was positively associated and Ch8 was negatively associated with MoCA-J total score. In exploratory subdomain analyses, Ch8 remained significantly associated with naming and attention scores. Nutritional and body composition analyses suggested a marginal negative association between fat intake and MoCA-J scores, whereas protein mass was positively associated with MoCA-J scores.

**Discussion:**

fNIRS-derived prefrontal Oxy-Hb responses during a seated motor–cognitive dual-task paradigm, together with nutritional and body composition indicators, may provide complementary information for characterizing cognitive health in community-dwelling older adults. Although exploratory and requiring longitudinal validation, this multidimensional assessment approach may contribute to practical community-based strategies for dementia prevention and healthy aging.

## 1 Introduction

The ability to perform dual tasks, which involves executing cognitive and motor tasks simultaneously, has been used as an indicator of age-related changes in cognitive and motor function (1). A decline in dual-task performance has been frequently reported in older adults (2, 3), reflecting age-related changes in attention and executive function (1). Most previous studies have examined cognitive–motor interference using dual-task walking paradigms. Older adults exhibit slower gait speed and increased error rates when performing cognitive tasks such as conversation, verbal fluency, or serial subtraction while walking, and these changes are exacerbated by aging and cognitive impairment (4–9). A decline in dual-task gait performance is associated with increased risks of falls, cognitive decline, and dementia (10), and longitudinal studies have suggested that dual-task gait performance may be useful for predicting dementia progression in patients with mild cognitive impairment (MCI) (11, 12).

However, seated motor-cognitive dual-task paradigms have also been developed as safer and more feasible alternatives for older adults, particularly in community-based settings. Previous studies using seated stepping exercise under dual-task conditions reported that this paradigm could be performed safely indoors because it reduces the risk of falling while still requiring simultaneous motor and cognitive processing (13). Another study developed a DVD-based seated dual-task stepping exercise in which participants performed a verbal fluency task while stepping as quickly as possible, demonstrating the feasibility of seated stepping combined with a cognitive task in older adults (14). These findings suggest that seated dual-task stepping can serve as a practical paradigm for assessing cognitive-motor interference when walking-based assessment is difficult or potentially unsafe.

The present study therefore used a seated stepping task combined with a verbal task. Although seated stepping does not reproduce the full biomechanical demands of walking, it retains the essential dual-task requirement of coordinating a repetitive motor task with concurrent cognitive processing. Accordingly, seated dual-task stepping is expected to engage executive control processes that are also relevant to gait-based dual-task performance, particularly those associated with prefrontal cortical function. This rationale is also consistent with previous intervention research showing that movement music therapy involving rhythmic physical movement with a percussion instrument and cognitive engagement improved physical and frontal lobe function in older adults with MCI (15). Thus, a seated motor-cognitive dual-task paradigm may be a feasible and safe approach for examining prefrontal activation related to cognitive-motor control in community-dwelling older adults.

Functional near-infrared spectroscopy (fNIRS) enables noninvasive measurement of changes in oxygenated and deoxygenated hemoglobin and allows evaluation of brain activity during motor and cognitive tasks under relatively naturalistic conditions (16–18). Previous fNIRS studies have shown that prefrontal oxygenated hemoglobin (Oxy-Hb) responses increase during dual-task performance compared with single-task performance, reflecting greater cognitive demands and the involvement of attention and executive control processes (12, 19–22). In older adults, increased prefrontal activation during dual-task performance may reflect compensatory recruitment or neural inefficiency, particularly among individuals with cognitive decline (12, 19, 20, 22). Therefore, measuring prefrontal Oxy-Hb responses during seated motor-cognitive dual-task performance may provide additional information regarding age-related cognitive and motor changes in community settings.

Recent reviews suggest that dual-task fNIRS assessment may be useful for examining task-related prefrontal Oxy-Hb responses in older adults, showing increased Prefrontal Oxy-Hb responses even in the absence of overt performance deterioration (10, 20, 22). Some longitudinal studies have suggested that altered prefrontal Oxy-Hb responses may be associated with later cognitive changes; however, further evidence is needed to establish predictive validity. fNIRS allows researchers and clinicians to capture subtle brain function differences in ecologically valid contexts, such as walking (11). However, comparisons across studies are complicated by differences in task protocols and measurement standards, and thus a consensus is needed regarding optimal dual-task conditions and outcome measures (11, 16). Moreover, most existing studies on this subject are small-scale cross-sectional investigations, highlighting the need for longitudinal studies to validate predictive validity. Finally, the interpretation of PFC overactivation—whether as compensation, inefficiency, or network disruption—requires caution, underscoring the importance of combining fNIRS with other biomarkers while considering individual differences and technical factors (11).

Cognitive decline in older adults is associated not only with cognitive test performance but also with changes in frontal lobe function, physical function, and nutritional status. The prefrontal cortex plays a key role in executive processing, and age-related changes in frontal activation may reflect task-dependent changes in cognitive control, compensatory recruitment, or reduced neural efficiency (23). fNIRS has been used to examine frontal lobe hemodynamics during cognitive, motor, and dual-task performance in older adults, including those with mild cognitive impairment, and may be useful for detecting task-related changes in frontal activation in this population. In addition, cognitive function in older adults with mild cognitive impairment has been associated with frailty, nutritional status, and quality of life, supporting the importance of multidimensional assessment in community and preventive care settings (24). Therefore, combining cognitive screening, neurophysiological assessment, and nutritional evaluation may be useful for characterizing possible cognitive decline in community-dwelling older adults.

Therefore, this study aimed to explore the feasibility of combining prefrontal Oxy-Hb responses measured using fNIRS during a seated motor–cognitive dual-task paradigm with cognitive screening and nutritional evaluation as a multidimensional community-based assessment of possible cognitive decline among older adults attending community salons.

## 2 Materials and methods

### 2.1 Study design and participants

This cross-sectional study was conducted among community-dwelling older adults between October 2022 and December 2023. Data were collected from participants recruited from local community centers and health salons during the same period. Inclusion criteria were: (1) age ≥65 years, (2) ability to walk without the use of walking aids, and (3) consent to participate in the study. Exclusion criteria included: (1) diagnosis of dementia or other neurological disorders, (2) severe cardiovascular or musculoskeletal conditions that could interfere with safe participation, or (3) inability to complete cognitive assessments. Written informed consent was obtained from all participants prior to data collection, and the study was carried out in accordance with the principles of the Declaration of Helsinki. The study protocol was approved by the Ethics Committee of Toyama Prefectural University (Approval No. KANGO DAI R4-15).

### 2.2 Measurements

#### 2.2.1 Cognitive function

Global cognitive function was assessed using the Montreal Cognitive Assessment (MoCA), which is widely used for screening MCI (25). Additionally, the Mini-Mental State Examination (MMSE) was administered to assess general cognitive status (26).

#### 2.2.2 Physical and nutritional status

Anthropometric measures including body mass index (BMI), body fat percentage, and skeletal muscle mass were obtained using a bioelectrical impedance analysis device with an InBody 470 body composition analyzer (InBody Co., Ltd., Seoul, Korea). Nutritional status was evaluated using the Food Frequency Questionnaire (short-FFQ) (27).

#### 2.2.3 Montreal cognitive assessment–Japanese version

The MoCA was originally developed as a brief screening tool to evaluate multiple cognitive domains, including attention, visuospatial ability, memory, language, conceptual thinking, calculation, and orientation, with a total score of 30 points. The MoCA has been translated into many languages, and the Japanese version (MoCA-J) has been validated (25).

MoCA-J scoring was conducted according to the standard scoring instructions for the Japanese version of the Montreal Cognitive Assessment. In accordance with these instructions, one point was added to the total score for participants with 12 years of education or fewer, while maintaining a maximum total score of 30 points. Therefore, the MoCA-J scores used in the present analyses were education-adjusted scores.

For the MoCA-J, a cutoff score of 26 has been reported as useful for screening possible MCI, with a sensitivity of 93% and a specificity of 87% (28). In the present study, this cutoff was used only as a screening criterion for possible cognitive decline, not as a clinical diagnosis of MCI.

### 2.3 Dual-task paradigm

The seated motor–cognitive dual-task paradigm consisted of seated stepping performed simultaneously with a verbal fluency task. The detailed fNIRS task sequence, including the seated stepping task, fixed vocalization control tasks, verbal fluency tasks, and the dual-task condition, is described.

### 2.4 fNIRS measurement protocol and analysis

#### 2.4.1 fNIRS measurement protocol

**Figure 1:**
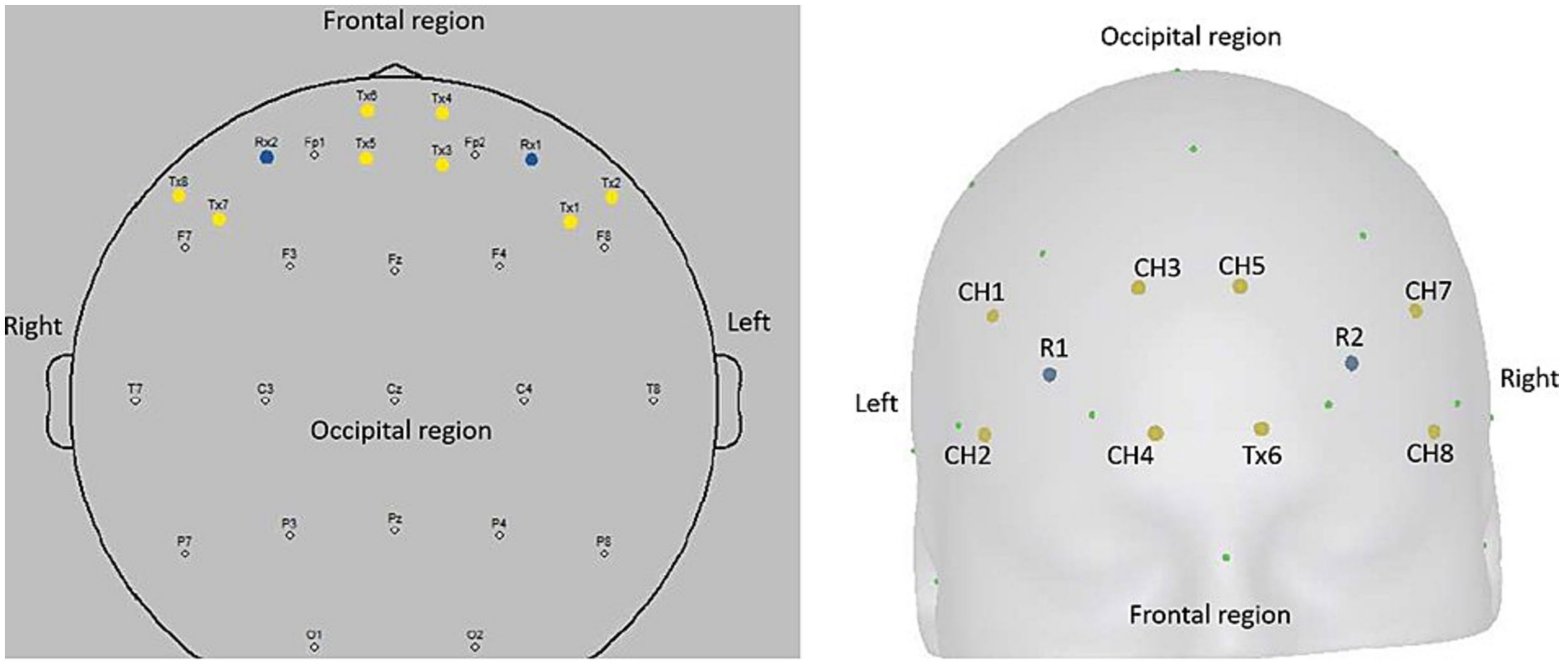
Approximate probe placement of the 8-channel fNIRS system over the prefrontal region. Yellow circles indicate transmitters and blue circles indicate receivers. Channel-to-cortex correspondence should be interpreted as approximate because individual three-dimensional digitization was not performed. Oxysoft device that was used, Artinis Medical Systems, Elst, https://artinis.com.

PFC activity was measured using a portable 8-channel fNIRS system (OctaMon, Artinis Medical Systems, The Netherlands). The system used two wavelengths, 765 and 855 nm, with an emitter–detector distance of 3.5 cm, corresponding to an estimated penetration depth of approximately 1.5–1.75 cm. Hemoglobin signals from the 8-channel system were recorded at a sampling rate of 10 Hz. Probe placement followed the manufacturer’s standard configuration for the prefrontal region, and the probe position digitization is shown in Figure 1. Because individual three-dimensional digitization was not performed, the anatomical localization of each channel should be interpreted as approximate.

**Figure 2:**
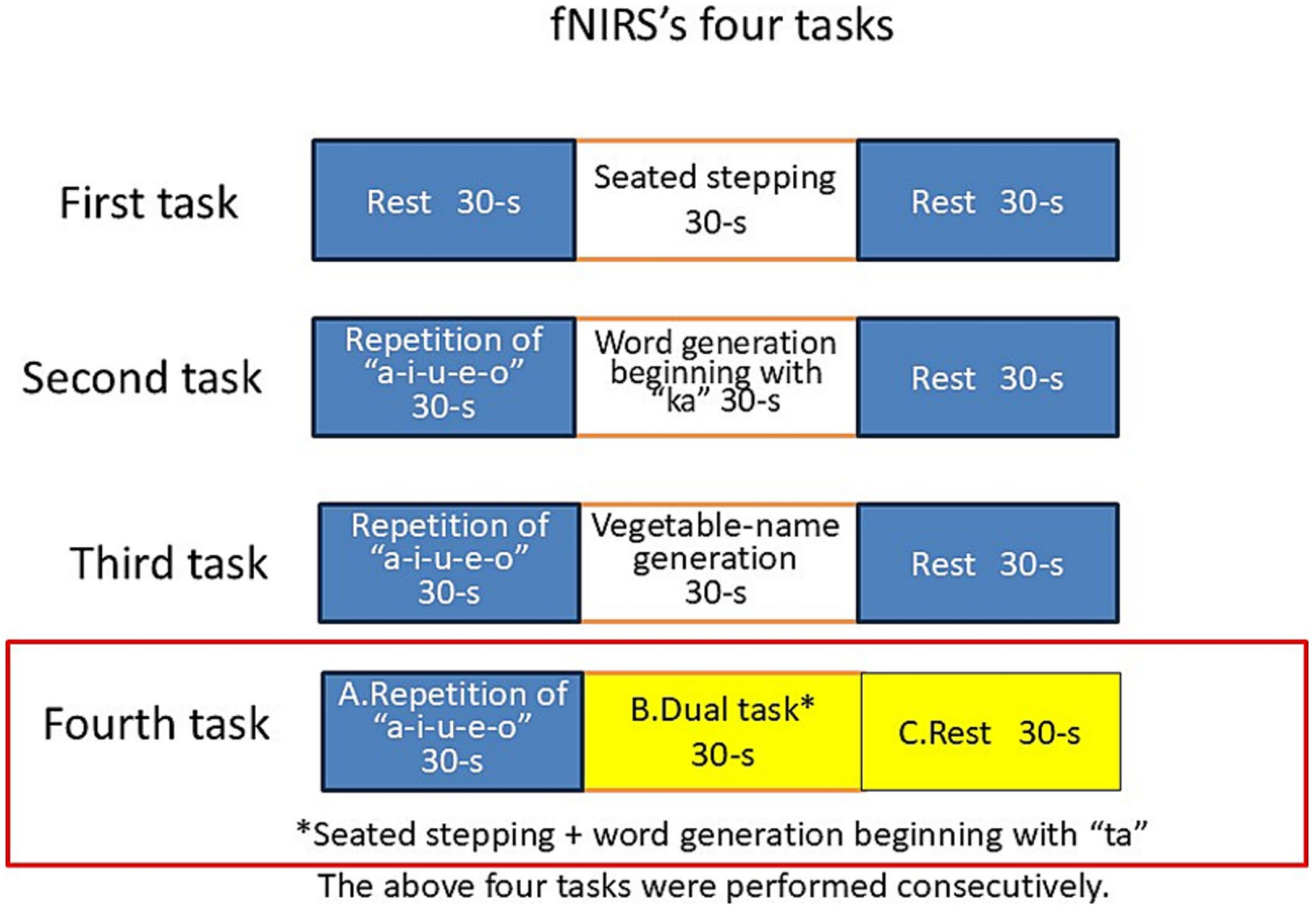
fNIRS task protocol. All tasks were performed while participants remained seated in a chair. The protocol consisted of four consecutive tasks: seated stepping, verbal fluency beginning with “ka,” vegetable-name generation, and a seated motor–cognitive dual task involving seated stepping with verbal fluency beginning with “ta.” Each verbal task was preceded by a 30-s fixed vocalization task in which participants repeatedly vocalized “a-i-u-e-o.” The integrated Oxy-Hb response during the dual-task condition was calculated from the 30-s dual-task period and the subsequent 30-s resting period, after subtracting the integrated Oxy-Hb value obtained during the immediately preceding 30-s fixed vocalization task.

The fNIRS task protocol was designed with reference to previous studies and consisted of four consecutive task conditions. All tasks were performed while participants remained seated in a chair. In the first task, participants rested for 30 s, performed seated stepping for 30 s, and then rested for 30 s. In the second task, participants repeatedly vocalized the Japanese vowels “a-i-u-e-o” for 30 s, followed by a verbal fluency task in which they generated as many words as possible beginning with the Japanese syllable “ka” for 30 s. This was followed by a 30-s resting period. In the third task, participants repeatedly vocalized “a-i-u-e-o” for 30 s, followed by a verbal fluency task in which they generated as many vegetable names as possible for 30 s. After another 30-s resting period, the fourth task was performed. In the fourth task, participants repeatedly vocalized “a-i-u-e-o” for 30 s, followed by the dual-task condition, in which they performed seated stepping while simultaneously generating as many words as possible beginning with the Japanese syllable “ta” for 30 s. The protocol ended with a final 30-s resting period (Figure 2).

Each task condition was performed once to minimize physical and cognitive burden among older participants and to maintain feasibility in community salon settings. All participants completed the protocol under the same standardized conditions.

#### 2.4.2 fNIRS data analysis and preprocessing

fNIRS data were analyzed using Oxysoft (Artinis Medical Systems, The Netherlands). Preprocessing included band-pass filtering with cutoff frequencies of 0.04 and 0.7 Hz to reduce baseline drift and cardiac pulsation. Motion artifacts were visually inspected in Oxysoft, and the data were checked for obvious transient spikes or discontinuities before analysis. No dedicated automated motion-artifact correction algorithm, such as wavelet filtering or spline interpolation, was applied. In addition, short-separation channels were not available in the present OctaMon configuration; therefore, systemic physiological correction using short-separation regression was not performed. Global signal filtering was also not applied.

A 30-s resting baseline window was used for the integrated Oxy-Hb calculation. Oxy-Hb changes during each task were calculated relative to the resting baseline period immediately preceding the task. For the dual-task analysis, the integrated Oxy-Hb value was calculated using the 30-s dual-task period in the fourth task and the subsequent 30-s resting period. The post-task resting period was included because the hemodynamic response to the dual task was expected to persist with a delay after task completion.

To reduce the influence of vocalization-related activation, the integrated Oxy-Hb value during the 30-s fixed vocalization task, in which participants repeatedly vocalized “a-i-u-e-o,” was subtracted from the integrated Oxy-Hb value obtained during the dual-task period and subsequent rest. The resulting value was used in the analysis as the integrated Oxy-Hb response during the dual task.

To evaluate prefrontal neural activity, Oxy-Hb was analyzed for each channel (CH1–CH8). Oxy-Hb was used as the primary fNIRS outcome because it is commonly reported in cognitive fNIRS studies and is considered sensitive to task-related cortical hemodynamic changes. Since HbR and total-Hb were not included in the main analyses, the physiological interpretation of the prefrontal Oxy-Hb responses should be made cautiously.

### 2.5 Outcome measures

The primary outcome was the integrated Oxy-Hb response in the PFC during the seated motor–cognitive dual-task condition. The integrated Oxy-Hb response during the dual task was defined as the value obtained during the 30-s dual-task period in the fourth task and the subsequent 30-s resting period. The post-task resting period was included because the hemodynamic response to the dual task was expected to persist with a delay after task completion.

To reduce the influence of hemodynamic changes associated with speech and articulation, the integrated Oxy-Hb value obtained during the 30-s fixed vocalization task immediately preceding the dual-task condition, in which participants repeatedly vocalized the Japanese vowels “a-i-u-e-o,” was subtracted from the integrated Oxy-Hb value obtained during the dual-task period and subsequent rest. The resulting value was used as the integrated Oxy-Hb response during the seated motor–cognitive dual-task condition.

Secondary outcomes included associations between PFC Oxy-Hb responses and MoCA-J total and subdomain scores, as well as nutritional, body composition, and biochemical variables, including food frequency questionnaire scores, BMI, skeletal muscle mass, protein mass, and lipid levels.

### 2.6 Statistical analysis

Descriptive statistics were performed for demographic, cognitive, nutritional, body composition, biochemical, and fNIRS variables. Continuous variables are presented as means and standard deviations or medians and interquartile ranges, as appropriate, and categorical variables as frequencies and percentages. Normality of continuous variables was assessed using the Shapiro–Wilk test. Pearson’s or Spearman’s correlation coefficients were calculated, depending on data distribution, to examine associations between total MoCA-J scores and Oxy-Hb responses in the eight prefrontal cortex channels during the seated motor-cognitive dual-task condition. Additional correlation analyses were performed to assess associations between MoCA-J scores and nutritional intake, body composition, and biochemical variables, including FFQ-derived dietary variables, BMI, body fat percentage, body water, protein mass, body weight, skeletal muscle mass, fat mass, and lipid profiles.

Exploratory hierarchical multiple regression analyses were conducted to examine associations between MoCA-J total and subdomain scores and Oxy-Hb responses across all eight fNIRS channels. The dependent variables were the MoCA-J total score and MoCA-J subdomain scores. Age and sex were entered as covariates in the first block, and all eight fNIRS channels were entered simultaneously in the second block using the forced-entry method. Because specific fNIRS channels were not pre-specified *a priori*, these channel-wise regression analyses were considered exploratory.

For the MoCA-J total score, detailed coefficient estimates are presented in the main table. For the MoCA-J subdomain scores, detailed coefficient estimates are presented for the main subdomain models, and a summary table is provided for all subdomain analyses, including delayed recall and orientation. Complete-case analysis was used for regression models; participants with missing fNIRS or covariate data were excluded from the relevant analyses.

Additional exploratory regression analyses were conducted to examine nutritional and body composition predictors of MoCA-J scores. To account for multiple comparisons, *p*-values for the eight fNIRS channel predictors within each regression model were adjusted using the Benjamini–Hochberg false discovery rate procedure. Age and sex were not included in the FDR correction. Both unadjusted and FDR-adjusted *p*-values are reported where appropriate.

MoCA-J scores were calculated according to the standard scoring instructions for the Japanese version and were education-adjusted at the scoring stage. However, detailed educational attainment was not collected; education was recorded only as a dichotomous variable, ≤12 years versus >12 years. Therefore, education could not be included as a continuous or more detailed covariate in the regression models. Statistical significance was set at two-tailed *p* < 0.05. All analyses were conducted using IBM SPSS Statistics version 28.0 (IBM Corp., Armonk, NY, United States).

## 3 Results

### 3.1 Participant characteristics and MoCA-J screening results

A total of 127 participants (36 males) with a mean age of 76.9 ± 6.5 years (range: 65–91) were included in the study. The mean BMI was 23.3 ± 3.0 (Table 1). Blood test results showed a mean random blood glucose, HbA1c, and 25-hydroxyvitamin D (25OHD) levels of 139.4 ± 38.5 mg/dL, 5.84 ± 0.59%, and 18.5 ± 5.6 ng/mL, respectively (Table 1). Regarding blood lipids, mean total cholesterol, triglycerides, high density lipoprotein (HDL), and low density lipoprotein (LDL) were 202.8 ± 37.3 mg/dL, 185.8 ± 122.4 mg/dL, 57.6 ± 14.6 mg/dL, and 116.7 ± 30.0 mg/dL, respectively. Psychosocial indicators included a mean Geriatric Depression Scale score of 3.1 ± 3.4 and a mean social isolation score of 16.1 ± 5.9 (Table 1).

**Table 1:** Basic characteristics of the participants (*n* = 127).

Variable	Mean ± SD	Min	Max
Age (years)	76.87 ± 6.50	65.00	91.06
MMSE	27.74 ± 2.22	21.00	30.00
MoCA	23.76 ± 3.91	10.00	30.00
GDS	3.13 ± 3.44	0.00	15.00
Social isolation score	16.05 ± 5.92	0.00	30.00
BMI	23.34 ± 3.01	16.40	34.00
Blood glucose (mg/dL)	139.38 ± 38.53	79.00	250.00
HbA1c (%)	5.84 ± 0.59	5.10	8.20
25OHD (ng/mL)	18.54 ± 5.55	5.20	30.90
Total cholesterol (mg/dL)	202.83 ± 37.32	121.00	310.00
Triglycerides (mg/dL)	185.78 ± 122.37	45.00	785.00
HDL cholesterol (mg/dL)	57.58 ± 14.61	32.00	120.00
LDL cholesterol (mg/dL)	116.71 ± 29.96	47.00	202.00

BMI, body mass index; HDL, high density lipoprotein; LDL, low density lipoprotein; MMSE, Mini-Mental State Examination; MoCA, Montreal Cognitive Assessment–Japanese version; GDS, geriatric depression score; HbA1c, glycated hemoglobin; 25OHD, 25-hydroxyvitamin D, Min, Minimum; Max, Maximum.

**Figure 3:**
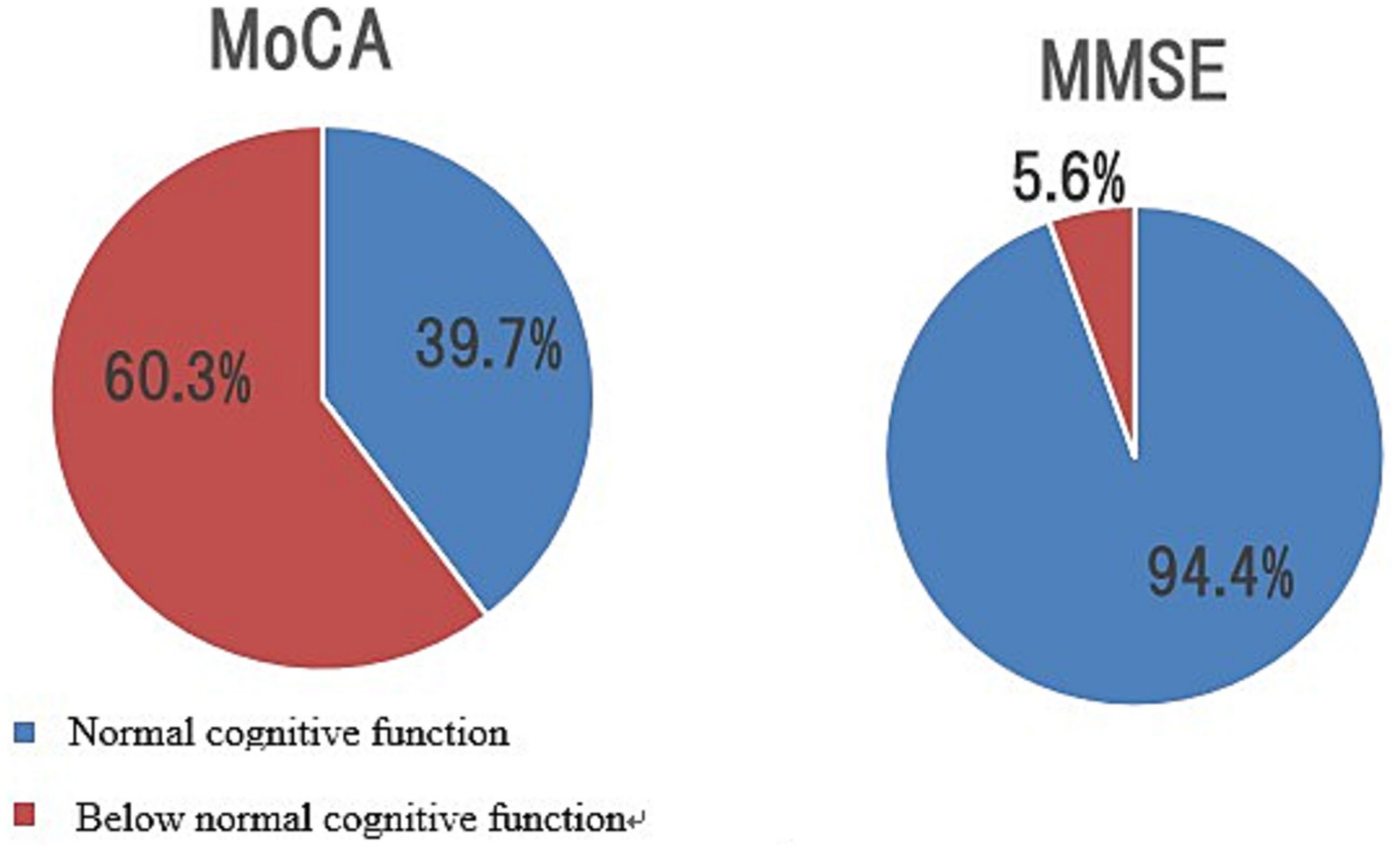
Cognitive function among community-dwelling older adults: MoCA-J: 39.7% within normal range, 60.3% below cutoff (possible MCI). MMSE: 94.4% within normal range, 5.6% below cutoff. MoCA-J, Montreal Cognitive Assessment-Japanese version; MMSE, Mini-Mental State Examination.

Cognitive assessment showed a mean MMSE score of 27.7 ± 2.22, with only 5.6% of participants below the cutoff. In contrast, the mean MoCA-J score was 23.7 ± 3.9, with approximately 60.3% of participants scoring below the cutoff of 26 (Table 1; Figure 3). These results indicate that nearly two-thirds of participants scored below the MoCA-J screening cutoff, suggesting possible cognitive decline rather than a clinical diagnosis of MCI (Figure 3).

### 3.2 Associations between MoCA-J scores and prefrontal hemodynamics

Exploratory hierarchical multiple regression analyses were conducted to examine the associations between MoCA-J scores and prefrontal Oxy-Hb responses across the eight fNIRS channels. Age and sex were entered as covariates in the first block, and all eight fNIRS channels were entered simultaneously in the second block using the forced-entry method. The *p*-values for the eight fNIRS channel predictors were adjusted using the Benjamini–Hochberg false discovery rate procedure. Complete fNIRS and covariate data were available for 114 participants, who were included in the regression analyses.

For the MoCA-J total score, the overall model was significant, *F*(10, 103) = 6.626, *p* < 0.001, explaining 39.1% of the variance. The addition of the eight fNIRS channels significantly improved the model beyond age and sex, ΔR^2^ = 0.139, F change (8, 103) = 2.937, *p* = 0.005. Among the fNIRS channels, Ch6 was positively associated with MoCA-J total score after FDR correction (*β* = 0.345, *p* = 0.004, FDR-adjusted *p* = 0.016), whereas Ch8 was negatively associated with MoCA-J total score after FDR correction (*β* = −0.375, *p* < 0.001, FDR-adjusted *p* < 0.003; Table 2).

**Table 2:** Multiple linear regression analysis of MoCA total score with age, sex, and Oxy-Hb values across 8 fNIRS channels.

Dependent variable: MoCA total score							
Predictor	B	SE	β	t	*p*	FDR-adjusted p	95% CI for B
Age	−0.285	0.049	−0.498	−5.832	<0.001	—	−0.382 to −0.188
Sex	0.697	0.699	0.083	0.998	0.321	—	−0.689 to 2.083
Ch1:	0.000	0.001	0.069	0.508	0.612	0.754	−0.001 to 0.002
Ch2:	0.000	0.001	0.051	0.414	0.680	0.754	−0.001 to 0.001
Ch3:	0.000	0.000	−0.095	−0.888	0.376	0.754	−0.001 to 0.000
Ch4:	0.000	0.001	0.042	0.333	0.740	0.754	−0.001 to 0.001
Ch5:	0.000	0.001	0.035	0.314	0.754	0.754	−0.001 to 0.002
Ch6:	0.002	0.001	0.345	2.968	0.004	0.016	0.001 to 0.003
Ch7:	0.000	0.001	0.047	0.376	0.708	0.754	−0.001 to 0.002
Ch8:	−0.001	0.000	−0.375	−3.674	<0.001	<0.003	−0.002 to −0.001

Model fit: R = 0.626, R^2^ = 0.391, adjusted R^2^ = 0.332, F(10, 103) = 6.626, *p* < 0.001. Incremental contribution of fNIRS channels after age and sex: ΔR^2^ = 0.139, F change (8, 103) = 2.937, *p* = 0.005. Regression analyses were based on complete cases with available fNIRS and covariate data (*n* = 114). B, unstandardized regression coefficient; SE, standard error; β, standardized regression coefficient; CI, confidence interval; FDR, false discovery rate. Age and sex were included as covariates. All eight fNIRS channels were entered simultaneously using the forced-entry method. FDR-adjusted *p*-values were calculated for the eight fNIRS channel predictors using the Benjamini–Hochberg procedure. Age and sex were not included in the FDR correction.

Exploratory regression analyses were also conducted for the MoCA-J subdomain scores using the same covariate structure. For visuospatial/executive function, the overall model was significant, *F*(10, 103) = 2.226, *p* = 0.022; however, the addition of the eight fNIRS channels did not significantly improve the model beyond age and sex, ΔR^2^ = 0.087, F change(8, 103) = 1.368, *p* = 0.219. No fNIRS channel remained statistically significant after FDR correction (Table 3A).

**Table 3A:** Multiple linear regression analysis for visuospatial/executive function.

Dependent variable: Visuospatial/Executive function score							
Predictor	B	SE	β	t	*p*	FDR-adjusted p	95% CI for B
Age	−0.036	0.015	−0.242	−2.443	0.016	—	−0.065 to −0.007
Sex	−0.304	0.208	−0.142	−1.458	0.148	—	−0.717 to 0.109
Ch1:	6.63 × 10^−5^	0.000	0.050	0.317	0.752	0.865	0.000 to 0.000
Ch2:	−5.24 × 10^−5^	0.000	−0.045	−0.310	0.757	0.865	0.000 to 0.000
Ch3:	−7.23 × 10^−6^	0.000	−0.007	−0.055	0.956	0.956	0.000 to 0.000
Ch4:	−6.57 × 10^−5^	0.000	−0.055	−0.377	0.707	0.865	0.000 to 0.000
Ch5:	9.75 × 10^−5^	0.000	0.062	0.475	0.636	0.865	0.000 to 0.001
Ch6:	0	0.000	0.276	2.043	0.044	0.176	0.000 to 0.001
Ch7:	0	0.000	0.080	0.550	0.584	0.865	0.000 to 0.001
Ch8:	0	0.000	−0.326	−2.753	0.007	0.056	−0.001 to 0.000

Model fit: R = 0.422, R^2^ = 0.178, adjusted R^2^ = 0.098, F(10, 103) = 2.226, *p* = 0.022. Incremental contribution of fNIRS channels after age and sex: ΔR^2^ = 0.087, F change(8, 103) = 1.368, *p* = 0.219.

For naming, the overall model was significant, *F*(10, 103) = 2.537, *p* = 0.009. The addition of the eight fNIRS channels did not significantly improve the model beyond age and sex, ΔR^2^ = 0.084, F change (8, 103) = 1.353, *p* = 0.226. However, Ch8 was negatively associated with naming score after FDR correction (*β* = −0.351, *p* = 0.003, FDR-adjusted *p* = 0.024; Table 3B).

**Table 3B:** Multiple linear regression analysis for naming.

Dependent variable: Naming score							
Predictor	B	SE	β	t	*p*	FDR-adjusted p	95% CI for B
Age	−0.03	0.008	−0.347	−3.542	<0.001	—	−0.047 to −0.013
Sex	0	0.120	0.000	0.004	0.997	—	−0.238 to 0.239
Ch1:	3.00 × 10^−5^	0.000	0.039	0.249	0.804	0.804	0.000 to 0.000
Ch2:	7.40 × 10^−5^	0.000	0.108	0.758	0.450	0.662	0.000 to 0.000
Ch3:	−7.70 × 10^−5^	0.000	−0.124	−1.016	0.312	0.662	0.000 to 0.000
Ch4:	6.65 × 10^−5^	0.000	0.095	0.661	0.510	0.662	0.000 to 0.000
Ch5:	0	0.000	0.122	0.944	0.347	0.662	0.000 to 0.000
Ch6:	0	0.000	0.142	1.060	0.292	0.662	0.000 to 0.000
Ch7:	−6.84 × 10^−5^	0.000	−0.080	−0.556	0.579	0.662	0.000 to 0.000
Ch8:	0	0.000	−0.351	−2.999	0.003	0.024	0.000 to 0.000

Model fit: R = 0.445, R^2^ = 0.198, adjusted R^2^ = 0.120, F(10, 103) = 2.537, *p* = 0.009. Incremental contribution of fNIRS channels after age and sex: ΔR^2^ = 0.084, F change (8, 103) = 1.353, *p* = 0.226.

For attention, the overall model was significant, *F*(10, 103) = 3.470, *p* < 0.001. The addition of the eight fNIRS channels showed a marginal but non-significant improvement beyond age and sex, ΔR^2^ = 0.110, F change(8, 103) = 1.887, *p* = 0.070. Ch8 was negatively associated with attention score after FDR correction (*β* = −0.386, *p* < 0.001, FDR-adjusted *p* < 0.003; Table 3C).

**Table 3C:** Multiple linear regression analysis for attention.

Dependent variable: Attention score							
Predictor	B	SE	β	t	*p*	FDR-adjusted p	95% CI for B
Age	−0.054	0.014	−0.359	−3.789	<0.001	—	−0.083 to −0.026
Sex	−0.196	0.205	−0.089	−0.954	0.342	—	−0.603 to 0.211
Ch1:	0	0	0.143	0.955	0.342	0.684	0.000 to 0.001
Ch2:	1.69 × 10^−5^	0	0.014	0.102	0.919	0.935	0.000 to 0.000
Ch3:	0	0	−0.19	−1.607	0.111	0.296	0.000 to 0.000
Ch4:	−4.60 × 10^−5^	0	−0.037	−0.268	0.789	0.935	0.000 to 0.000
Ch5:	−1.67 × 10^−5^	0	−0.01	−0.082	0.935	0.935	0.000 to 0.000
Ch6:	0	0	0.212	1.645	0.103	0.296	0.000 to 0.001
Ch7:	0	0	0.091	0.653	0.515	0.824	0.000 to 0.001
Ch8:	0	0	−0.386	−3.41	<0.001	<0.003	−0.001 to 0.000

Model fit: R = 0.502, R^2^ = 0.252, adjusted R^2^ = 0.179, F(10, 103) = 3.470, *p* < 0.001. Incremental contribution of fNIRS channels after age and sex: ΔR^2^ = 0.110, F change (8, 103) = 1.887, *p* = 0.070.

For language, the overall model was significant, *F*(10, 103) = 2.146, *p* = 0.027; however, the addition of the eight fNIRS channels did not significantly improve the model beyond age and sex, ΔR^2^ = 0.082, F change(8, 103) = 1.283, *p* = 0.261. No fNIRS channel remained statistically significant after FDR correction (Table 3D).

**Table 3D:** Multiple linear regression analysis for language score.

Dependent variable: Language score							
Predictor	B	SE	β	t	*p*	FDR-adjusted p	95% CI for B
Age	−0.039	0.013	−0.296	−2.970	0.004	—	−0.065 to −0.013
Sex	−0.082	0.187	−0.043	−0.437	0.663	—	−0.453 to 0.289
Ch1:	0.000	0.000	0.225	1.433	0.155	0.320	0.000 to 0.001
Ch2:	0.000	0.000	0.112	0.774	0.441	0.541	0.000 to 0.000
Ch3:	0.000	0.000	−0.176	−1.415	0.160	0.320	0.000 to 0.000
Ch4:	0.000	0.000	−0.105	−0.720	0.473	0.541	0.000 to 0.000
Ch5:	7.28 × 10^−6^	0.000	0.005	0.039	0.969	0.969	0.000 to 0.000
Ch6:	0.000	0.000	0.228	1.680	0.096	0.320	0.000 to 0.001
Ch7:	0.000	0.000	−0.118	−0.802	0.424	0.541	−0.001 to 0.000
Ch8:	0.000	0.000	−0.252	−2.116	0.037	0.296	0.000 to 0.000

Model fit: R = 0.415, R^2^ = 0.172, adjusted R^2^ = 0.092, F(10, 103) = 2.146, *p* = 0.027. Incremental contribution of fNIRS channels after age and sex: ΔR^2^ = 0.082, F change (8, 103) = 1.283, *p* = 0.261.

For abstraction, the overall model was not significant, F(10, 103) = 1.631, *p* = 0.108. The addition of the eight fNIRS channels did not significantly improve the model beyond age and sex, ΔR^2^ = 0.101, F change (8, 103) = 1.505, *p* = 0.165. No fNIRS channel remained statistically significant after FDR correction (Table 3E).

**Table 3E:** Multiple linear regression analysis for abstraction score.

Dependent variable: Abstraction score							
Predictor	B	SE	β	t	*p*	FDR-adjusted p	95% CI for B
Age	0.006	0.008	0.079	0.772	0.442	—	−0.010 to 0.022
Sex	0.206	0.116	0.176	1.765	0.081	—	−0.025 to 0.436
Ch1:	6.72 × 10^−5^	0.000	0.093	0.576	0.566	0.755	0.000 to 0.000
Ch2:	6.80 × 10^−5^	0.000	0.106	0.719	0.474	0.755	0.000 to 0.000
Ch3:	1.68 × 10^−5^	0.000	0.029	0.229	0.819	0.849	0.000 to 0.000
Ch4:	0.000	0.000	−0.258	−1.742	0.085	0.331	0.000 to 0.000
Ch5:	7.62 × 10^−5^	0.000	0.089	0.665	0.508	0.755	0.000 to 0.000
Ch6:	0.000	0.000	0.215	1.549	0.124	0.331	0.000 to 0.000
Ch7:	2.27 × 10^−5^	0.000	0.029	0.191	0.849	0.849	0.000 to 0.000
Ch8:	0.000	0.000	−0.236	−1.946	0.054	0.331	0.000 to 0.000

Model fit: R = 0.370, R^2^ = 0.137, adjusted R^2^ = 0.053, F(10, 103) = 1.631, *p* = 0.108. Incremental contribution of fNIRS channels after age and sex: ΔR^2^ = 0.101, F change (8, 103) = 1.505, *p* = 0.165.

A summary of the exploratory regression analyses for all MoCA-J subdomain scores, including delayed recall and orientation, is shown in Table 4. Across the subdomain models, the addition of the eight fNIRS channels did not significantly improve model fit beyond age and sex. After Benjamini–Hochberg FDR correction across the eight channel predictors within each model, Ch8 remained significantly associated with naming and attention scores, whereas no fNIRS channel remained significantly associated with visuospatial/executive function, language, abstraction, delayed recall, or orientation scores. These findings should be interpreted as exploratory and hypothesis-generating.

**Table 4:** Summary of exploratory regression analyses for MoCA-J subdomain scores.

Outcome	R	R^2^	Adjusted R^2^	ΔR^2^ for fNIRS channels	F change	p for F change	fNIRS channels significant after FDR correction
Visuospatial/executive function	0.422	0.178	0.098	0.087	1.368	0.219	None
Naming	0.445	0.198	0.120	0.084	1.353	0.226	Ch8
Attention	0.502	0.252	0.179	0.110	1.887	0.070	Ch8
Language	0.415	0.172	0.092	0.082	1.283	0.261	None
Abstraction	0.370	0.137	0.053	0.101	1.505	0.165	None
Delayed recall	0.602	0.363	0.301	0.061	1.230	0.289	None
Orientation	0.279	0.078	−0.012	0.045	0.622	0.758	None

MoCA-J, Japanese version of the Montreal Cognitive Assessment; fNIRS, functional near-infrared spectroscopy; FDR, false discovery rate. Hierarchical multiple regression analyses were conducted for each MoCA-J subdomain score. Age and sex were entered as covariates in the first block, and all eight fNIRS channels were entered simultaneously in the second block using the forced-entry method. ΔR^2^ indicates the additional variance explained by the eight fNIRS channels beyond age and sex. FDR-adjusted *p*-values were calculated for the eight fNIRS channel predictors within each model using the Benjamini–Hochberg procedure. Age and sex were not included in the FDR correction. Detailed coefficient estimates are presented in Supplementary Tables S1, S2.

### 3.3 Nutritional intake, body composition, and cognitive function

Correlation analyses showed positive associations between MoCA-J scores and several body composition and biochemical variables. Body water, *r* = 0.306, *p* = 0.002; protein mass, *r* = 0.317, *p* = 0.002; and body weight, *r* = 0.274, *p* = 0.006, were positively correlated with MoCA-J scores. Triglyceride levels showed a weak positive correlation with MoCA-J scores, *r* = 0.188, *p* = 0.042. Total cholesterol showed a marginal association, *r* = 0.175, *p* = 0.055. No significant associations were observed for BMI, body fat percentage, skeletal muscle mass, fat mass, HDL cholesterol, or LDL cholesterol (Table 5).

**Table 5:** Correlations between MoCA-J score and body composition/biochemical variables.

Variable	Unit	Pearson’s r	*p*-value
BMI	kg/m^2^	0.050	0.326
Body fat percentage	%	−0.081	0.230
Body water	%	0.306	0.002**
Protein mass	kg	0.317	0.002**
Body fat	kg	0.062	0.286
Body weight	kg	0.274	0.006**
Skeletal muscle mass	kg	−0.113	0.153
Fat mass	kg	0.023	0.417
Total cholesterol	mg/dL	0.175	0.055†
Triglycerides	mg/dL	0.188	0.042*
HDL cholesterol	mg/dL	0.095	0.194
LDL cholesterol	mg/dL	0.137	0.106

MoCA-J, Japanese version of the Montreal Cognitive Assessment; BMI, body mass index; HDL, high-density lipoprotein; LDL, low-density lipoprotein. †*p* < 0.10, *p < 0.05, **p < 0.01.

After considering multiple comparisons across body composition and biochemical variables, the associations with body water, protein mass, and body weight remained the most robust findings, whereas the association with triglyceride levels should be interpreted cautiously because of its small effect size and borderline significance.

Body composition analysis indicated a mean body fat percentage of 28.3 ± 7.9%, body water of 30.4 ± 6.0 L, and skeletal muscle mass of 16.2 ± 8.5 kg. Nutritional intake analyses showed mean daily energy consumption of 1979.5 ± 241.6 kcal and protein, fat, and carbohydrate intakes of 71.4 ± 20.4 g, 54.5 ± 14.4 g, and 235.4 ± 60.5 g, respectively. Micronutrient intake included 3000.2 ± 955.8 mg of potassium, 611.2 ± 241.1 mg of calcium, and 16.2 ± 5.4 g of dietary fiber (Table 6).

**Table 6:** Descriptive statistics.

Variable	Mean ± SD	Min	Max
Body fat percentage (%)	28.28 ± 7.86	9.10	46.00
Body water (%)	30.40 ± 5.98	18.30	45.20
Body fat mass (kg)	16.45 ± 5.84	4.60	35.70
Skeletal muscle mass (kg)	16.16 ± 8.46	4.01	34.10
Fat mass (kg)	16.53 ± 4.98	4.60	27.20
Energy intake (kcal)	1979.50 ± 241.62	1708.00	2869.00
Protein (g)	71.41 ± 20.43	34.30	128.30
Fat (g)	54.48 ± 14.37	25.10	83.00
Saturated fatty acids (g)	16.03 ± 4.79	6.68	29.92
Carbohydrates (g)	235.37 ± 60.45	109.10	394.00
Potassium (mg)	3000.17 ± 955.80	1171.00	5710.00
Dietary fiber (g)	16.19 ± 5.43	5.80	45.40
Calcium (mg)	611.18 ± 241.06	220.00	1789.00

Exploratory regression analyses were then performed to examine nutritional and body composition predictors of MoCA-J scores. In the regression model using FFQ-derived nutritional variables, fat intake showed a marginal negative association with MoCA-J scores, *β* = −0.05, *t* = −2.02, unadjusted *p* = 0.050, R^2^ = 0.07, adjusted R^2^ = 0.06. However, because multiple FFQ variables were examined and the association was at the threshold of significance, this finding should be interpreted as exploratory rather than confirmatory (Table 7).

**Table 7:** Linear regression analysis for total MoCA-J score using FFQ fat with multiple-comparison correction.

Predictor	Unstandardized β	SE	t	Unadjusted *p*-value	FDR significance after correction	95% CI for β	VIF
Constant	26.55	1.42	18.70	< 0.001	—	23.74 to 29.37	—
FFQ Fat	−0.05	0.03	−2.02	0.050	Not significant	−0.10 to 0.00	1.00

Model fit: R^2^ = 0.07, adjusted R^2^ = 0.06. The dependent variable was the total MoCA-J score. FFQ Fat was examined in the context of multiple FFQ-derived nutritional variables. Because the unadjusted p-value for FFQ Fat was 0.050, this association should be interpreted cautiously and did not remain statistically significant after considering multiple comparisons across FFQ variables. SE, standard error; CI, confidence interval; VIF, variance inflation factor; FFQ, Food Frequency Questionnaire; MoCA-J, Japanese version of the Montreal Cognitive Assessment. Variables entered: Energy (kcal), Potassium, Calcium, Protein, Saturated fatty acids, Carbohydrates, Dietary fiber. SE, standard error, CI, confidence interval; VIF, variance inflation factor; FFQ, Food Frequency Questionnaire.

In the regression model using body composition variables, protein mass was positively associated with MoCA-J scores, *β* = 0.65, *t* = 2.90, *p* < 0.001, R^2^ = 0.07, adjusted R^2^ = 0.06. This association was remained significant after accounting for multiple comparisons across body composition variables. However, the explanatory power of the model was limited, as indicated by the small R^2^ value. Therefore, the association between protein mass and MoCA-J scores should be interpreted as an exploratory association rather than evidence of a causal or predictive relationship (Table 8).

**Table 8:** Linear regression analysis for total MoCA-J score using body composition variables with multiple-comparison correction.

Predictor	Unstandardized *β*	SE	t	Unadjusted *p*-value	FDR significance after correction	95% CI for β	VIF
Constant	18.73	1.83	10.22	< 0.001	—	15.10 to 22.36	—
Protein mass (kg)	0.65	0.22	2.90	< 0.001	Significant	0.21 to 1.10	1.00

Model fit: R^2^ = 0.07, adjusted R^2^ = 0.06. The dependent variable was the total MoCA-J score. Protein mass was examined in the context of multiple body composition variables, including BMI, body fat percentage, body water, body fat, body weight, and protein mass. Multiple-comparison correction was considered using the Benjamini–Hochberg false discovery rate procedure. Because the unadjusted p-value for protein mass was < 0.001, this association was considered to remain statistically significant after FDR correction. SE, standard error; CI, confidence interval; VIF, variance inflation factor; FDR, false discovery rate; MoCA-J, Japanese version of the Montreal Cognitive Assessment. CI, confidence interval; SE, standard error; VIF, variance inflation factor; MoCA, Montreal Cognitive Assessment-Japanese version.

## 4 Discussion

### 4.1 Possible cognitive decline based on MoCA-J screening among community-dwelling older adults

In this study, approximately 60% of community-dwelling older adults scored below the MoCA-J cutoff value. This finding should not be interpreted as the prevalence of MCI, but rather as the proportion of participants who showed possible cognitive decline based on MoCA-J screening. Previous reports have indicated that MoCA is more sensitive than MMSE in detecting mild cognitive decline (25, 29). Epidemiological studies have shown that the prevalence of MCI varies widely from 0.5 to 42%, depending on the region and survey method (30). The relatively high proportion observed in the present study may reflect the characteristics of the community salon participants, selection bias, and limitations of applying a single cutoff value to a heterogeneous older population.

A high proportion of participants scoring below the MoCA-J cutoff was observed in both rural and urban community salons, suggesting that possible cognitive decline based on screening was not limited to a specific geographical area. Although the present study did not directly examine contextual factors such as transportation access, medical resources, social isolation, or community ties, these factors may partly explain regional differences in cognitive health and should be examined in future studies.

According to projections, the prevalence of dementia in Japan will exceed 20% in many prefectures by the 2030s and 25% by 2035 (31). In response, the Japanese government has placed “prevention” alongside “coexistence” as core pillars of its National Dementia Strategy. However, current community-based preventive activities are often centered on recreation and social interaction, without systematic assessment of cognitive or physical function. While such activities are meaningful, their effectiveness in improving cognition remains unclear.

Incorporating objective outcome measures such as MoCA, body composition assessment, and brain activity measured by fNIRS into preventive programs may help maintain motivation among older adults and provide visible evidence of intervention benefits (32–34). Establishing a system for continuous assessment and feedback would allow older adults to better understand their health condition, experience tangible benefits, and maintain engagement in preventive programs.

### 4.2 Relationship between brain activation and cognitive function: potential role of fNIRS as an adjunctive assessment tool

In this study, MoCA-J scores were positively associated with prefrontal Oxy-Hb responses measured using fNIRS during a seated motor-cognitive dual-task condition. These findings suggest that fNIRS-derived prefrontal Oxy-Hb responses may capture aspects of cognitive function that are not fully reflected by conventional cognitive screening scores alone. Although the observed associations were modest, the results indicate that prefrontal hemodynamic responses may provide supplementary information for multidimensional assessment of cognitive health in community-dwelling older adults. In fNIRS studies, Oxy-Hb responses are widely used as an index of task-related cortical activation. Previous studies have reported that prefrontal Oxy-Hb responses increase during cognitive or motor-cognitive dual-task performance and are associated with executive function and cognitive processing (17, 18, 33, 34). The present findings are consistent with this literature and suggest that even a feasible seated motor-cognitive task conducted in community settings may elicit prefrontal Oxy-Hb responses related to cognitive screening performance.

An important strength of the present study is that fNIRS measurement was conducted in a community salon setting rather than in a highly controlled laboratory environment. fNIRS is portable, non-invasive, and suitable for assessing brain activity during relatively naturalistic tasks. Therefore, combining fNIRS-derived prefrontal Oxy-Hb responses with MoCA-J and nutritional/body composition assessment may offer a practical approach to characterizing cognitive health among older adults who participate in community-based preventive activities. At the same time, the findings should be interpreted cautiously. The present study was cross-sectional, and behavioral dual-task performance indices, such as verbal fluency word counts, stepping cadence, and dual-task cost, were not included. Therefore, the observed Oxy-Hb responses should not be interpreted as direct evidence of compensatory recruitment, neural inefficiency, or gait-cognition coupling. Rather, they should be understood as exploratory associations between cognitive screening scores and task-related prefrontal hemodynamic responses. Future longitudinal studies incorporating both neural and behavioral measures are needed to clarify the functional and predictive significance of fNIRS-derived prefrontal Oxy-Hb responses.

These results support the feasibility of incorporating neurophysiological assessment into community-based cognitive health programs, while highlighting the need for careful interpretation and further validation.

### 4.3 Exploratory channel-level associations between MoCA-J scores and prefrontal oxy-Hb responses

The present exploratory analyses showed that MoCA-J total score was associated with prefrontal Oxy-Hb responses in Ch6 and Ch8 after adjustment for age and sex and Benjamini–Hochberg FDR correction. In the subdomain analyses, Ch8 remained significantly associated with naming and attention scores, whereas no fNIRS channel remained significantly associated with visuospatial/executive function, language, abstraction, delayed recall, or orientation scores after FDR correction. These findings suggest that task-related prefrontal Oxy-Hb responses during a seated motor–cognitive dual-task condition may provide supplementary information related to cognitive screening performance in community-dwelling older adults.

This channel-level perspective may be useful for community-based cognitive health assessment because conventional screening tools such as the MMSE and MoCA-J provide information about global and domain-specific cognitive performance, but do not directly capture task-related neurophysiological responses. By adding fNIRS-derived Oxy-Hb responses, it may be possible to obtain additional information about prefrontal hemodynamic responses during cognitively demanding activities. Such information may help characterize cognitive health more comprehensively in community-dwelling older adults and may also provide objective and visible feedback that could support motivation for participation in preventive programs.

Nevertheless, the present channel-level findings should be interpreted with appropriate caution. The fNIRS channels were not pre-specified *a priori*, and the analyses were exploratory. Moreover, the anatomical correspondence between fNIRS channels and cortical regions was approximate because the probe placement followed the manufacturer’s standard configuration and individual three-dimensional digitization was not performed. Therefore, the observed channel-level associations should not be regarded as evidence of distinct regional cortical specialization or causal neural mechanisms. Future studies using more precise anatomical registration, repeated task trials, behavioral performance indices, and larger samples are needed to confirm and extend these findings.

### 4.4 Effects of dietary habits and body composition on cognitive function

The present findings should be interpreted within the context of research suggesting that cognitive function in older adults is related to multiple domains, including frontal lobe function, physical function, and nutritional status. Age-related changes in prefrontal activation may reflect task-dependent changes in cognitive control, compensatory recruitment, or reduced neural efficiency, particularly during cognitively demanding tasks (23). In addition, fNIRS has been used to examine frontal lobe hemodynamics during cognitive, motor, and dual-task performance in older adults, including those with mild cognitive impairment, and may provide useful information regarding task-related changes in frontal activation. Nutritional and physical factors may also be relevant to cognitive function in older adults; cognitive function in individuals with mild cognitive impairment has been reported to be associated with frailty, nutritional status, and quality of life (24). Therefore, the present study extends this multidimensional perspective to community settings by combining MoCA-J, fNIRS-derived prefrontal Oxy-Hb responses during a seated motor–cognitive dual-task paradigm, nutritional intake, and body composition indicators in older adults attending community salons.

The analyses suggested exploratory associations between nutritional/body composition indicators and cognitive function. Higher fat intake was associated with lower MoCA scores, whereas greater protein mass was significantly correlated with higher MoCA scores. Prior studies have shown that excessive intake of saturated fatty acids increases the risk of Alzheimer’s disease and MCI (35), whereas protein-balanced dietary patterns contribute to the maintenance of cognitive function (32).

High-fat diets have been linked to vascular dysfunction, insulin resistance, and amyloid-*β* deposition, all of which contribute to cognitive decline (36). In contrast, protein intake provides essential amino acids necessary for neurotransmitter synthesis and may contribute to cognitive resilience (37). The positive association between protein mass and MoCA scores in this study suggests that maintaining muscle reserves through adequate protein intake may be protective against cognitive decline, consistent with reports that sarcopenia is a risk factor for poorer cognitive outcomes (38). In the Japanese dietary context, this finding requires nuanced interpretation. Traditional Japanese diets are relatively low in total fat but include significant amounts of fish-derived polyunsaturated fatty acids, which have been associated with protective effects on cognitive health. In contrast, recent dietary shifts toward Western-style patterns, characterized by higher intake of animal fats and processed foods, may contribute to the observed negative impact of fat intake on cognition. Differentiating between the effects of saturated fats and polyunsaturated fatty acids will therefore be important for tailoring culturally appropriate dietary recommendations in dementia prevention strategies.

Taken together, these findings indicate that both dietary habits and body composition play important roles in the maintenance of cognitive function in community-dwelling older adults. Fat intake showed only a marginal negative association with MoCA-J scores, whereas protein mass showed a positive association. These findings should be interpreted cautiously because of the cross-sectional design and small explanatory power of the models. From a preventive perspective, community-based interventions should integrate dietary education and nutritional assessment with cognitive and physical evaluations. Combining dietary guidance, body composition monitoring, and fNIRS-based assessment of brain activity could provide a comprehensive framework for dementia prevention in aging societies.

### 4.5 Community salon as a platform for dementia prevention

Our findings suggest that community salons may serve as feasible venues for multidimensional assessment and preventive support related to cognitive health. In Japan, community salons are recognized within the framework of the national dementia strategy and the community-based integrated care system. These salons are widely operated by municipalities and volunteer organizations and are accessible, safe spaces where older adults can gather regularly. However, most current salon activities focus on social participation and recreation, without incorporating systematic health assessments. The integration of objective measures such as MoCA-J screening, fNIRS-based assessment of prefrontal Oxy-Hb responses, and nutritional evaluation into salon programs may strengthen their role as venues for cognitive health assessment and preventive support.

### 4.6 Integration with municipal initiatives and community general support centers

Municipal governments in Japan are increasingly required to implement evidence-based programs that support healthy aging and reduce long-term care burden. We provide a practical model for incorporating multidimensional assessments into existing municipal salon activities. Importantly, collaboration with Community General Support Centers—established under the long-term care insurance system to provide comprehensive support for older adults—could ensure that findings from salon-based screenings are effectively linked to appropriate follow-up, including referrals, health guidance, and family support. Since fNIRS is portable, non-invasive, and relatively cost-effective compared to other neuroimaging modalities, it can be feasibly deployed in community settings by trained health professionals, such as public health nurses. Coupled with nutritional assessment and body composition monitoring, these tools could allow municipalities to provide enjoyable social activities, deliver health monitoring and personalized preventive guidance.

### 4.7 Strengthening the community-based integrated care system

By incorporating fNIRS-based assessment of prefrontal Oxy-Hb responses and dietary evaluation into salon programs in collaboration with municipal authorities and Community General Support Centers, Japan’s community-based integrated care system can be further strengthened. Such integration would help identify older adults who may benefit from further assessment or preventive support who may not seek clinical assessment, promote multidisciplinary collaboration among public health nurses, nutritionists, and community volunteers, and contribute to community-level efforts to support cognitive health and healthy aging. Furthermore, this approach aligns with Japan’s national dementia policy, which emphasizes prevention, coexistence, and community-level support.

### 4.8 Limitations

This study has several limitations. First, because of the cross-sectional design, causal relationships and temporal ordering among cognitive function, prefrontal Oxy-Hb responses, and nutritional/body composition indicators cannot be determined. Therefore, the present findings do not establish whether altered prefrontal Oxy-Hb responses precede cognitive decline or reflect compensatory recruitment or neural inefficiency. Longitudinal studies are needed to clarify the predictive validity and mechanistic significance of fNIRS-derived measures.

Second, the explanatory power of the regression models was limited. The coefficients of determination were small, with R^2^ values ranging from 0.04 to 0.07. Thus, the observed associations should be interpreted as exploratory rather than diagnostic or predictive. In addition, participants were limited to older adults attending community salons in specific regions, which may restrict the generalizability of the findings and introduce selection bias.

Third, detailed information on educational attainment was not collected. Education was recorded only as a dichotomous variable, ≤12 years versus >12 years, and therefore could not be included as a continuous or more detailed covariate in the regression models. Although the MoCA-J includes a + 1 point adjustment for individuals with lower educational attainment, this scoring adjustment does not fully substitute for statistical adjustment for education. This should be considered a limitation of the present study.

Fourth, several methodological limitations related to fNIRS measurement should be considered. The probe placement followed the manufacturer’s standard configuration for the prefrontal region; however, precise channel coordinates and channel-to-cortex correspondence were not available, and individual three-dimensional digitization was not performed. Therefore, the anatomical localization of each fNIRS channel should be interpreted as approximate, and the observed channel-level associations should not be regarded as evidence of precise cortical localization. Each task condition was performed only once to reduce physical and cognitive burden among older participants, which may have limited the reliability of task-related fNIRS estimates. In addition, the present analysis focused on Oxy-Hb, and HbR and total-Hb were not included in the main analyses. No short-separation channels or additional global physiological filtering were used; therefore, systemic physiological influences could not be fully separated from cortical hemodynamic responses. Future studies should use more detailed anatomical registration, repeated trials, comprehensive reporting of Oxy-Hb, HbR, and total-Hb, and improved correction methods for systemic physiological noise.

Fifth, behavioral performance indices during the dual-task condition were not assessed. Specifically, verbal fluency word counts, stepping cadence, and dual-task cost were not measured. Therefore, the functional meaning of prefrontal Oxy-Hb responses cannot be fully determined, and the findings should not be interpreted as evidence of compensatory recruitment, neural inefficiency, or gait-cognition coupling. Furthermore, although the repeated “a-i-u-e-o” vocalization task was used to control, at least in part, for articulation and speech production, it did not fully control for lexical retrieval, semantic processing, selection, or inhibitory processes involved in verbal fluency. Future studies should include behavioral performance measures and additional speech-matched control tasks, such as automatic counting, to better distinguish speech-related hemodynamic changes from cognitive task-related activation.

Overall, the present findings suggest exploratory associations among cognitive screening scores, prefrontal Oxy-Hb responses, and nutritional/body composition indicators in community-dwelling older adults. These findings may support the potential value of multidimensional assessment in community salon settings, but they do not establish diagnostic, predictive, or mechanistic utility.

## Data Availability

The original contributions presented in the study are included in the article/Supplementary material, further inquiries can be directed to the corresponding author.
