# Supplementary material for: Multidimensional assessment of cognitive function in community-dwelling older adults: fNIRS dual-task assessment and nutritional evaluation in Japanese salons
Source: Front Public Health. 2026 Jun 30;14:1770437. doi: 10.3389/fpubh.2026.1770437 (PMC13364586; doi:10.3389/fpubh.2026.1770437)
Supplement: Supplementary file 1 [file Data_Sheet_1.docx]

**Supplementary Tables S1**

**Dependent variable: Delayed recall score**

| **Predictor** | **B** | **SE** | **β** | **t** | **p** | **FDR-adjusted p** | **95% CI for B** |
| --- | --- | --- | --- | --- | --- | --- | --- |
| Age | -0.123 | 0.022 | -0.487 | -5.570 | <.001 | — | -0.167 to -0.079 |
| Sex | 0.972 | 0.315 | 0.264 | 3.086 | .003 | — | 0.347 to 1.597 |
| Ch1: | 0.000 | 0.000 | -0.119 | -0.859 | .392 | .715 | -0.001 to 0.000 |
| Ch2: | 1.50 × 10⁻⁵ | 0.000 | 0.007 | 0.058 | .954 | .954 | 0.000 to 0.001 |
| Ch3: | 8.81 × 10⁻⁵ | 0.000 | 0.048 | 0.444 | .658 | .752 | 0.000 to 0.000 |
| Ch4: | 0.000 | 0.000 | 0.120 | 0.938 | .351 | .715 | 0.000 to 0.001 |
| Ch5: | 0.000 | 0.000 | -0.072 | -0.621 | .536 | .715 | -0.001 to 0.000 |
| Ch6: | 0.000 | 0.000 | 0.197 | 1.653 | .101 | .715 | 0.000 to 0.001 |
| Ch7: | 0.000 | 0.000 | 0.120 | 0.934 | .352 | .715 | 0.000 to 0.001 |
| Ch8: | 0.000 | 0.000 | -0.074 | -0.712 | .478 | .715 | 0.000 to 0.000 |

**Model fit:** R = .602, R² = .363, adjusted R² = .301, F(10, 103) = 5.866, p < .001.
**Incremental contribution of fNIRS channels after age and sex:** ΔR² = .061, F change(8, 103) = 1.230, p = .289.
