# Supplementary material for: Multidimensional assessment of cognitive function in community-dwelling older adults: fNIRS dual-task assessment and nutritional evaluation in Japanese salons
Source: Front Public Health. 2026 Jun 30;14:1770437. doi: 10.3389/fpubh.2026.1770437 (PMC13364586; doi:10.3389/fpubh.2026.1770437)
Supplement: Supplementary file 2 [file Data_Sheet_2.docx]

**Supplementary Tables S2**

**Dependent variable: Orientation score**

| **Predictor** | **B** | **SE** | **β** | **t** | **p** | **FDR-adjusted p** | **95% CI for B** |
| --- | --- | --- | --- | --- | --- | --- | --- |
| Age | -0.003 | 0.002 | -0.123 | -1.172 | .244 | — | -0.008 to 0.002 |
| Sex | -0.023 | 0.035 | -0.066 | -0.638 | .525 | — | -0.093 to 0.048 |
| Ch1: | 6.07 × 10⁻⁶ | 0.000 | 0.028 | 0.171 | .865 | .989 | 0.000 to 0.000 |
| Ch2: | -1.14 × 10⁻⁷ | 0.000 | -0.001 | -0.004 | .997 | .997 | 0.000 to 0.000 |
| Ch3: | -3.13 × 10⁻⁵ | 0.000 | -0.184 | -1.400 | .165 | .875 | 0.000 to 0.000 |
| Ch4: | -1.14 × 10⁻⁵ | 0.000 | -0.059 | -0.383 | .703 | .959 | 0.000 to 0.000 |
| Ch5: | 1.26 × 10⁻⁵ | 0.000 | 0.050 | 0.360 | .719 | .959 | 0.000 to 0.000 |
| Ch6: | 2.31 × 10⁻⁵ | 0.000 | 0.112 | 0.780 | .437 | .875 | 0.000 to 0.000 |
| Ch7: | 4.43 × 10⁻⁵ | 0.000 | 0.189 | 1.223 | .224 | .875 | 0.000 to 0.000 |
| Ch8: | -1.89 × 10⁻⁵ | 0.000 | -0.121 | -0.964 | .337 | .875 | 0.000 to 0.000 |

**Model fit:** R = .279, R² = .078, adjusted R² = -.012, F(10, 103) = 0.866, p = .567.
**Incremental contribution of fNIRS channels after age and sex:** ΔR² = .045, F change(8, 103) = 0.622, p = .758.

**Note.** B = unstandardized regression coefficient; SE = standard error; β = standardized regression coefficient; CI = confidence interval; FDR = false discovery rate. Age and sex were included as covariates. All eight fNIRS channels were entered simultaneously using the forced-entry method. FDR-adjusted p-values were calculated for the eight fNIRS channel predictors using the Benjamini–Hochberg procedure. Age and sex were not included in the FDR correction. Very small unstandardized coefficients are presented in scientific notation.
